# Supplementary material for: A Fluorescence Polarization-Based High-Throughput Screen to Identify the First Small-Molecule Modulators of the Human Adenylyltransferase HYPE/FICD
Source: Int J Mol Sci. 2020 Sep 27;21(19):7128. doi: 10.3390/ijms21197128 (PMC7582957; doi:10.3390/ijms21197128)
Supplement: Supplementary file 1 [file ijms-21-07128-s001.pdf]

International Journal of
Molecular Sciences

MDPI

Article

Supplementary Material

A fluorescence polarization-based high-throughput screen to identify the first small molecule modulators of the human adenylyltransferase HYPE/FICD

Ali Camara [1], Alyssa George [1$], Evan Hebner [1$], Anika Mahmood [1], Jashun Paluru [2] and Seema Mattoo [1,3,4,*]

[1] Department of Biological Sciences, Purdue University, West Lafayette, 47907, USA; acamara@purdue.edu (A.C.); george97@purdue.edu (A.G.); mahmooa@purdue.edu (A.M.); ehebner@purdue.edu (E.H.); smattoo@purdue.edu (S.M.)
[2] William Henry Harrison High School, West Lafayette, 47906, USA; jashpaluru@gmail.com (J.P.)
[3] Purdue Institute for Integrative Neuroscience, Purdue University, West Lafayette, 47907, USA
[4] Purdue Institute for Inflammation, Immunology and Infectious Disease, Purdue University, West Lafayette, 47907, USA
[$] These authors contributed equally
* Correspondence: smattoo@purdue.edu

Received: 27 August 2020; Accepted: 25 September 2020; Published: 27 September 2020

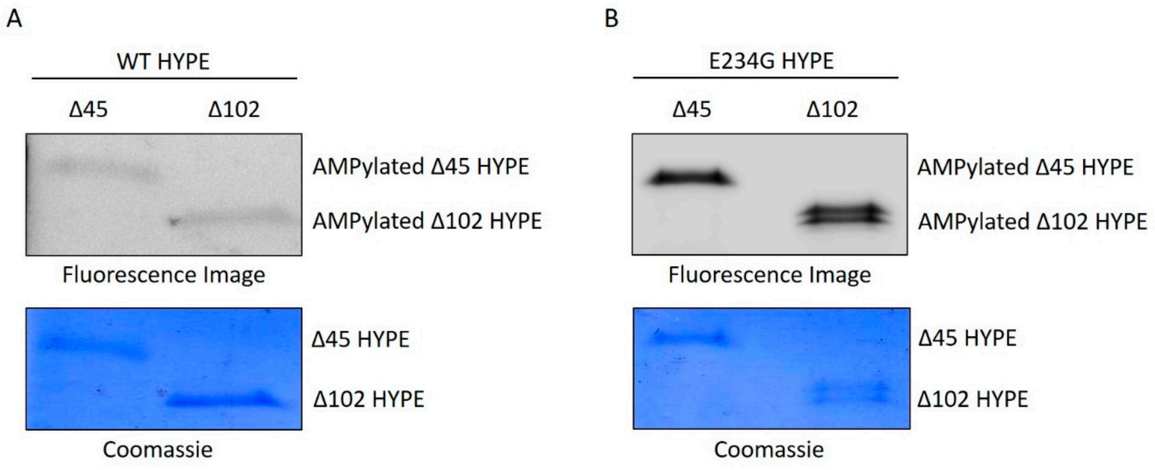

Figure S1 AutoAMPylation comparison between Δ45 and Δ102 HYPE. **A)** Fluorescence image (top) and corresponding Coomassie (bottom) showing side-by-side autoAMPylation reactions of Δ45 and Δ102 WT HYPE. **B)** As in A), but with E234G HYPE.

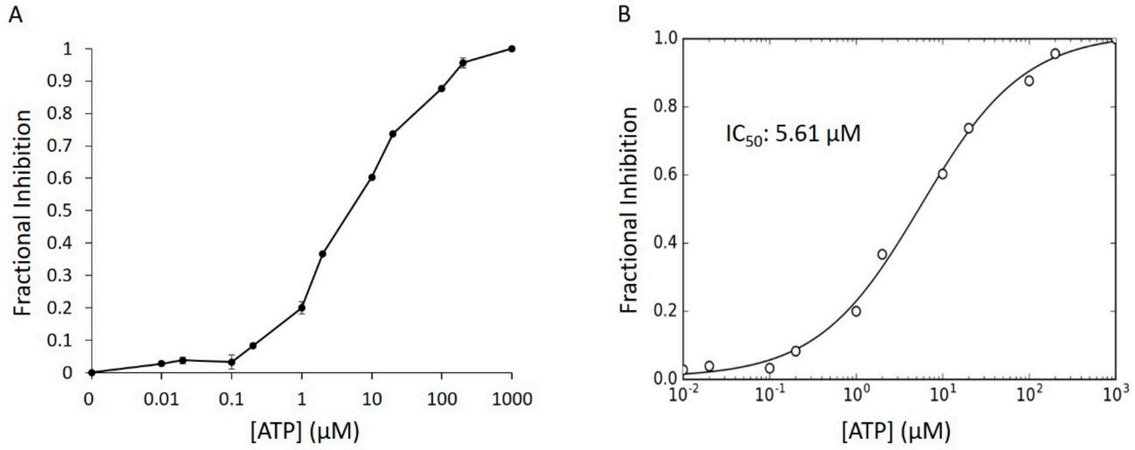

Figure S2 ATP inhibition of E234G HYPE FP autoAMPylation. **A)** Concentration-response curve assessing the inhibitory effect of unlabeled ATP on Δ102 E234G HYPE autoAMPylation using a constant Fl-ATP concentration. Data represented as the mean +/- SEM of three replicates. **B)** Data from A) plotted for IC_{50} value determination.

Table S1 WT HYPE HTS Hit Information

Library Identity	Number of HTS Hits*	HTS Hit Rate for Total Screen	HTS Hit Rate for Library
Total Screen	31	0.320%	---
LOPAC	6	0.062%	0.469%
Spectrum	7	0.072%	0.292%
MEGx	0	0.000%	0.000%
NATx	18	0.186%	0.360%

* Number of HTS hits is defined as all hits after manual correction for auto-fluorescent and fluorescence-quenching compounds.

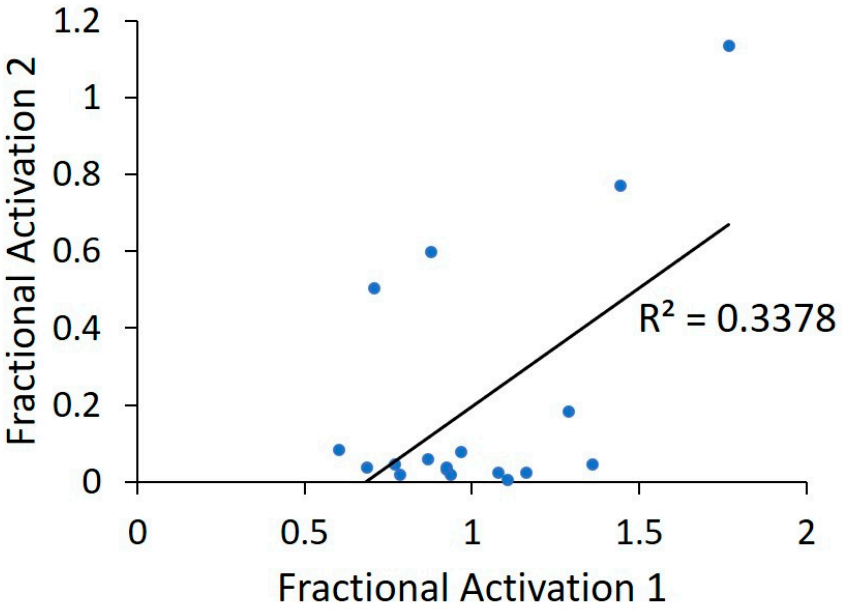

Figure S3 WT HYPE activator validation at 50% hit definition. Reprocessed data from Figure 6A to show Δ102 WT HYPE hit activity correlation between HTS (x-axis) and second-pass validation (y-axis) using the same FP autoAMPylation assay. Each dot represents the same compound incubated in two independent AMPylation reactions.

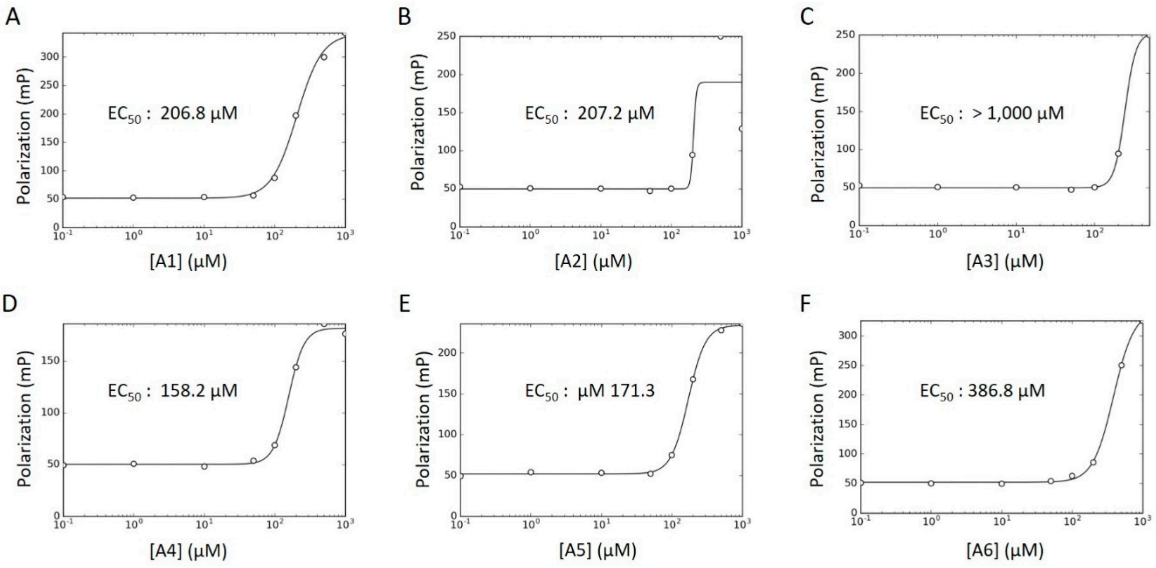

Figure S4 WT HYPE activator concentration-response curve. **A)-F)** FP plots from Δ102 WT HYPE AMPylation reactions incubated with 0 to 1,000 µM of DMSO-dissolved activators. All data were fitted to **Equation 5** (see Methods) to determine EC_{50} values. All reactions were done in buffer containing 0.1% Triton X-100.

Table S2 Hill's Coefficient for WT HYPE Activator

Compound	Triton X-100	No Triton X-100
A1	No	5.44
A1	Yes	2.43
A2	No	3.62
A2	Yes	21.40
A3	No	2.99
A3	Yes	6.29
A4	No	3.10
A4	Yes	3.97
A5	No	4.32
A5	Yes	3.57
A6	Yes	2.98

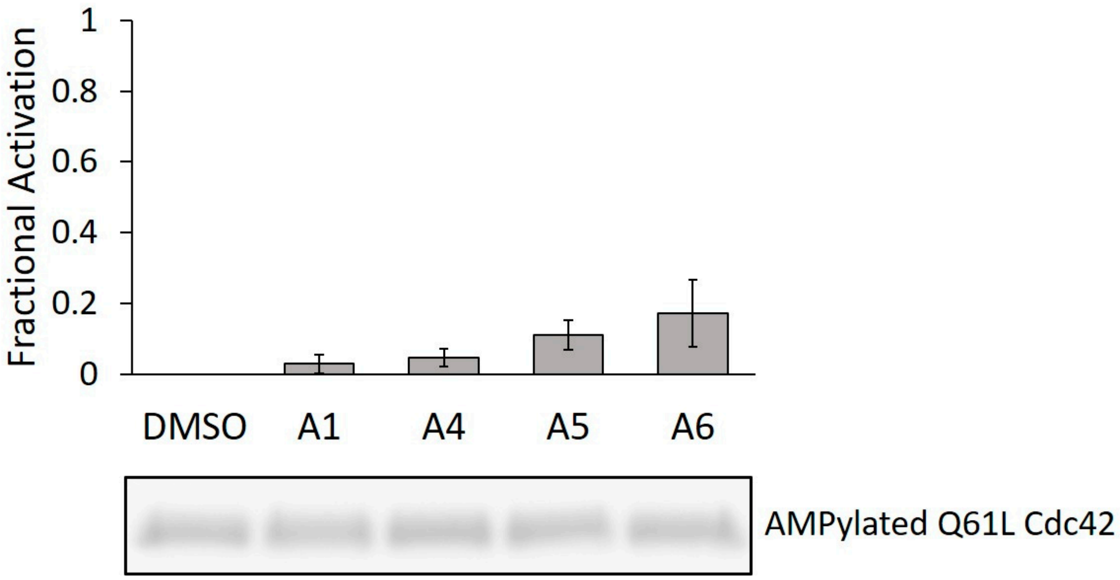

Figure S5 WT HYPE activator specificity assessment on IbpA-DR2-mediated AMPylation of Q61L Cdc42. In-gel fluorescence showing the quantification of three independent experiments (top) and representative fluorescence image (bottom). All reactions were performed in the presence of 0.1% Triton X-100. All activator samples were normalized to the DMSO control. Significance between activator samples and controls were determined by unpaired t-tests.

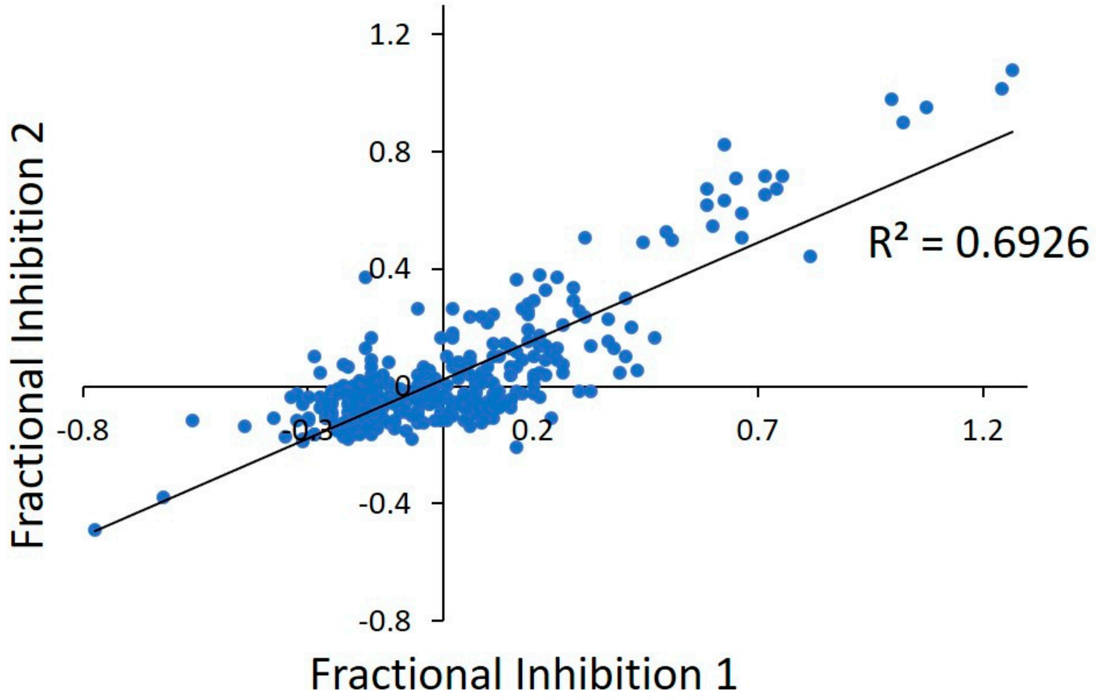

Figure S6 HTS assay reproducibility assessment. Duplicate plates containing the same 320 compounds each from the NATx library were simultaneously run with Δ102 E234G HYPE in an FP autoAMPylation assay. Reactions were run in minimal buffer without detergent and normalized to internal positive (Δ102 E234G HYPE) and negative (Δ102 WT HYPE) DMSO controls. Each dot represents the same compound incubated in two independent AMPylation reactions.

Table S3 E234G HYPE HTS Hit Information

Library Identity	Number of HTS Hits*	HTS Hit Rate for Total Screen	HTS Hit Rate for Library
Total Screen	95	0.981%	---
LOPAC	30	0.310%	2.34%
Spectrum	57	0.589%	2.38%
MEGx	0	0.000%	0.000%
NATx	8	0.083%	0.160%

* Number of HTS hits is defined as all hits after manual correction for auto-fluorescent and fluorescence-quenching compounds.

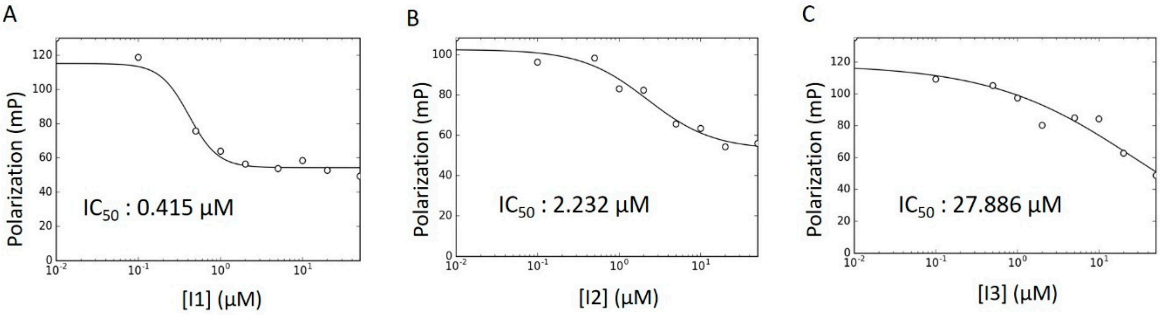

Figure S7 E234G HYPE inhibitor concentration-response curve. **A-C)** FP data from Δ102 E234G HYPE AMPylation reactions incubated with 0 to 200 μM of DMSO-dissolved inhibitors fitted to **Equation 6** (see Methods) to determine IC_{50} values. All reactions were done in buffer without detergent.

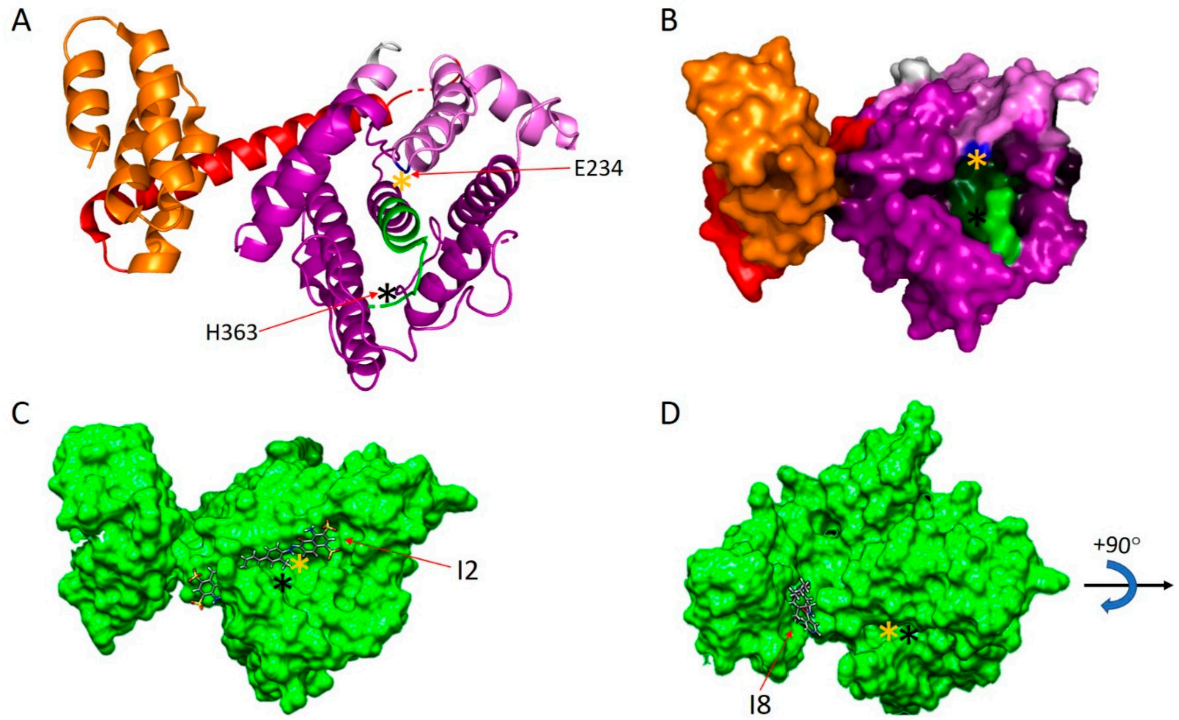

Figure S8 Molecular docking of HYPE and inhibitors. **A)** Ribbon crystal structure of HYPE (PDB: 4u04). The TPR domain is shown in orange, the linker region in red, the Fic domain in purple, the Fic motif in green, and the inhibitory glutamate (E234) in blue. Red arrows point to the inhibitory glutamate (yellow asterisk) and the catalytic histidine (H363, black asterisk). Image was visualized using Pymol software. **B)** Space-filling structure as in A). **C)** As in B), but with docked I2 compound (red arrow). Image was docking in SwissDock and visualized with Chimera. **D)** As in B) and C), but with I8 negative control compound. Structure is rotated 90° relative to B) for docking clarity.

Table S4 Molecular Docking Parameters for HYPE-Inhibitor Complexes

Docking Model	Optimal Predicted ΔG (J)	Recurring Models
I2	-3.52	5/5
I8	-6.21	1/5

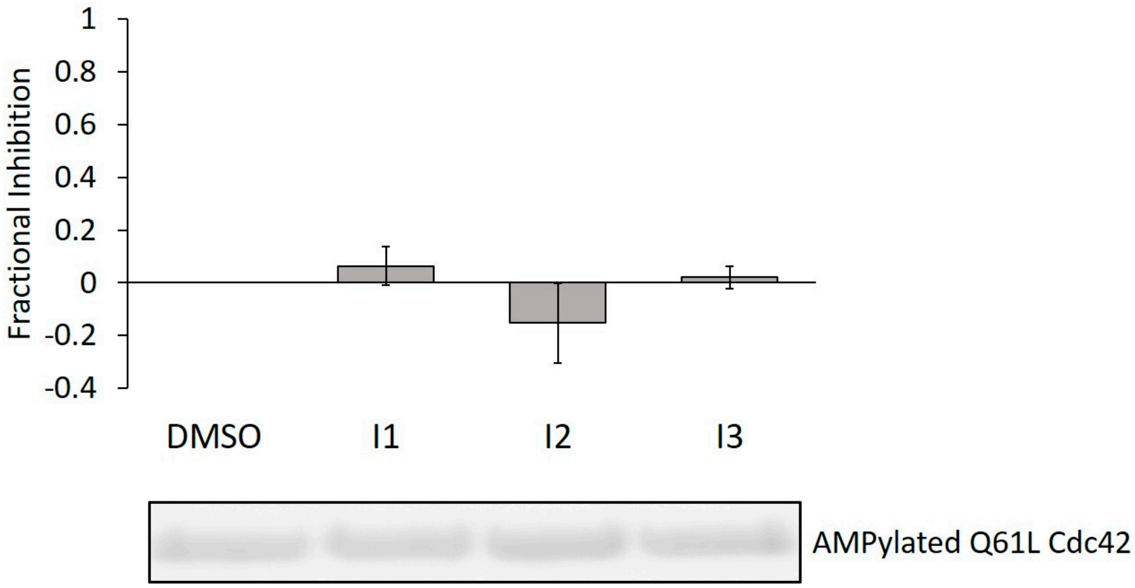

Figure S9 E234G HYPE inhibitor specificity assessment on IbpA-DR2-mediated AMPylation of Q61L Cdc42. In-gel fluorescence showing the quantification of three independent experiments (top) and representative fluorescence image (bottom). All reactions were performed in the presence of 0.1% Triton X-100. All inhibitor samples were normalized to the DMSO control. Significance between activator samples and controls were determined by unpaired t-tests.
